# New Insights on Hepatitis B Virus Viral Transcription in Single Hepatocytes

**DOI:** 10.3390/v16121828

**Published:** 2024-11-25

**Authors:** Bo Peng, Lixia Pan, Wenhui Li

**Affiliations:** 1 National Institute of Biological Sciences, Beijing 102206, China; panlixia@nibs.ac.cn; 2 Tsinghua Institute of Multidisciplinary Biomedical Research, Tsinghua University, Beijing 100084, China

**Keywords:** HBV, transcription, single cell

## Abstract

The hepatitis B virus (HBV) infects approximately 290 million people globally, with chronic infection sustained by persistent viral gene expression. Recent single-cell analyses of HBV viral transcripts have uncovered novel features of HBV transcription and provided fresh insights into its regulation at the single-cell level. In this review, we summarize the latest advancements in understanding HBV viral transcription in individual hepatocytes and highlight emerging technologies that hold promise for future research.

## 1. Introduction

The hepatitis B virus (HBV) is a 3.2 kb DNA virus, but its replication occurs through reverse transcription, which can be effectively inhibited by current nucleos(t)ide analogue (NUC) drugs. However, these drugs do not inhibit viral gene expression and, therefore, have no direct impact on viral antigenemia, an indicator of active infection [1,2,3]. A productive HBV infection relies on the active transcription of covalently closed circular (ccc) DNA, an episomal viral minichromosome located in the nucleus. This cccDNA, driven by four promoters and two enhancers, serves as the template for all viral transcripts. These include the 3.5 kb pregenomic (pg) RNA, which acts as a replication intermediate and codes for both the core protein and polymerase; the 3.5 kb precore (preC) RNA for HBV e antigen (HBeAg); the 2.4 kb preS1 RNA for the large envelope protein (L); the 2.1 kb preS2/S RNA for the medium (M) and small (S) envelope proteins; and the 0.7 kb RNA for HBV X protein (HBx) [4,5,6]. The presence of HBV surface antigen (HBsAg), especially alongside HBeAg in the blood, signifies active HBV infection [7].

The nonstructural HBx protein plays a crucial role in productive cccDNA transcription [8,9]. However, its exact mechanism of action remains under investigation. The best-known mechanism involves the induction of the degradation of the structural maintenance of the chromosome 5/6 (Smc5/6) complex, which releases transcriptional suppression mediated by the complex [10,11]. Inactivated cccDNA is not randomly distributed within the nucleus but appears to localize in specific regions [12,13].

An HBV-infected cell may contain several cccDNA molecules, which are highly compacted, with approximately two-thirds of the genome coding for at least two genes. All viral transcripts from a single cccDNA molecule share the same 3′ end [5]. These characteristics present technical challenges for distinguishing between viral transcripts using conventional analytical approaches. In recent years, the transcription start sites (TSSs) of HBV genes have been elucidated in greater detail using techniques such as full-length 5′RACE (rapid amplification of cDNA ends) and CAGE (cap analysis of gene expression) [14,15]. However, since these studies were based on bulk cell samples, they could not distinguish viral transcripts originating from different hepatocytes or determine the levels of viral transcripts from different cccDNA molecules within a single cell.

**Figure 1:**
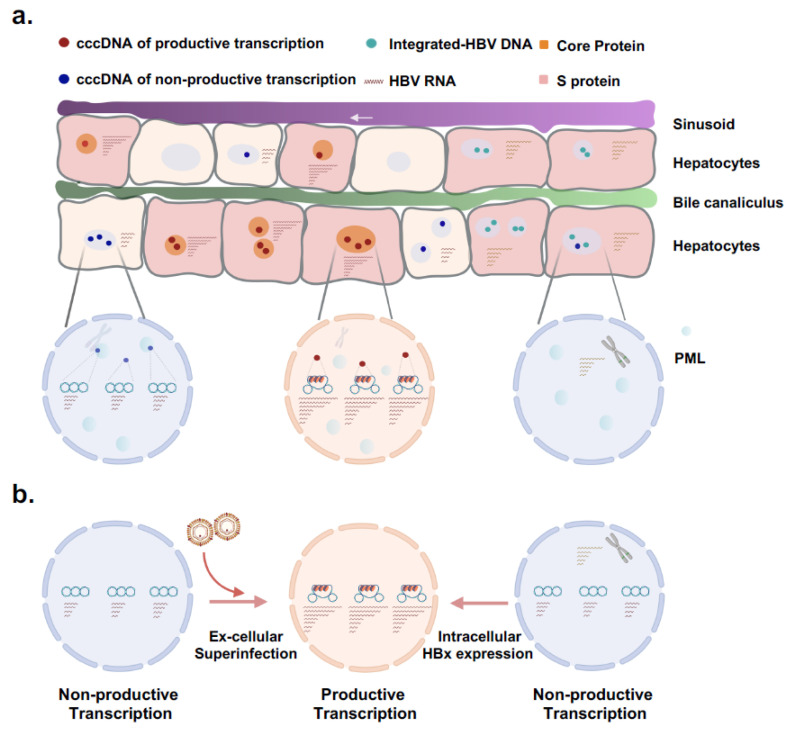
Schematics of hepatitis B virus viral transcription in single hepatocytes. (**a**) Heterogeneity of hepatocyte population in chronic HBV infection. Schematic of plates of hepatocytes, sinusoids, and bile canaliculi. Representative hepatocytes infected or not infected by HBV are shown. In hepatocytes containing productive cccDNA (red dots), viral RNAs coding for pgRNA, preCore, preS1, preS2/S, and various lengths of HBV X-related genes are generated, and the viral surface antigen and core protein are expressed. In cells harboring non-productive cccDNA (dark blue dots), only HBV X-related transcripts are produced. The non-productive or transcriptional inactive cccDNA appears not randomly distributed in host nucleus, with an apparent proximity preference to PML (promyelocytic leukeamia) bodies and/or heterochromatin regions like that from chromosome 19. In cells with no cccDNA but with integrated HBV DNA fragment(s) (cyan dots), depending on the integrated viral fragment and the integration site in the host genome, HBsAg and HBx, to a lesser extent, preS1-related transcripts are produced, and the viral proteins are expressed. Hepatocytes with HBV integration may undergo clone expansion. (**b**) Maintenance and reactivation of cccDNA transcription. Hepatocytes with productive viral transcription serve as the source of persistent infection in the liver. Hepatocytes are known to undergo continuous turnover usually within a time frame of 0.7 years for the diploid cells [24]. Over the course, the hepatocyte population with productive viral transcription from cccDNA is likely replenished by either de novo infection of naïve cells or reactivation of the transcription-inactive cccDNA within infected cells. Non-productive cccDNA can be activated through either super-infection or the expression of HBx from integrated HBV within infected cells.

Given that the liver contains hepatocytes with distinct functions and that there may be phenotype mixing (i.e., trans-complementation of variants) of viral infections within cells, it is crucial to understand viral transcription at the single-cell level. This understanding is key to developing antiviral strategies aimed at permanently silencing viral transcription. Recent advancements in single-cell sequencing and single-molecule imaging now allow transcriptome studies in thousands of individual cells simultaneously [16,17,18]. The emerging research, some of it on natural infections with or without drug treatments, using recently developed techniques such as droplet digital PCR (ddPCR), single-cell sequencing, and in situ hybridization, has uncovered important characteristics of HBV transcription at the single-cell level [19,20,21,22,23]. In this review, we summarize the recent progress and discuss the significance of these observations for HBV transcription in individual cells (Figure 1).

## 2. Viral Gene Expression Profiling

### 2.1. Transcription Initiation of HBV cccDNA

To characterize the HBV transcriptome at a single-cell resolution, Peng et al. utilized 5′ RNA sequencing and analyzed approximately 3000 hepatocytes isolated from liver-humanized FRG mice, which were stably infected with HBV and exhibited high levels of viremia. Although limited by the lack of longitudinal viral transcript data within single cells, that study suggested that the cccDNA may initiate transcription first within the X/Enhancer I domain in a stochastic manner. The accumulation of X transcripts, particularly those capable of translating functional X protein, might trigger the productive transcription of cccDNA. The transcription start site (TSS) for HBx was found near the first start codon (ATG) of the X open reading frame (ORF) [22]. Earlier studies by Stadelmayer et al., using full-length 5′ RACE profiling of HBV RNAs, also revealed diversity in HBx transcripts post-infection in HepG2-NTCP cells and primary human hepatocytes (PHHs) [14].

Peng et al. further showed that the transcription of HBx and related transcripts may proceed even in the presence of the Smc5/6 complex. By infecting PHHs with a virus bearing a stop codon mutation (residue 8) in HBx, they conducted 5′ single-cell sequencing 7 days after infection and found the majority of viral transcripts were from the HBx/enhancer I domain, suggesting that the initiation of HBx transcription occurs irrespective to the presence or absence of the SMC5/6 complex and before viral structural gene transcription [22]. The exact mechanism underlying HBx transcription remains unclear, though G-quadruplexes (G4s) in the HBx-related regions and nucleosome occupancy have been proposed to play a role [25,26]. Interestingly, HBx transcripts were detected as early as 4 h post-infection in PHHs and were maintained at comparable levels within the first 24 h, even when cccDNA was barely detectable [27]. Further studies are needed to determine whether any HBx transcripts are enclosed within the viral nucleocapsid. In vivo, liver cells exhibit transcriptome-wide and proteomic zonation [28,29], but whether liver zonation—characterized by the distinct expression of liver transcription factors in different zones—affects HBV transcription is largely unknown.

### 2.2. Coordinated HBV cccDNA Transcription in Single Cells

HBV transcripts can originate from both cccDNA and integrated viral DNA fragments [30,31]. As mentioned earlier, HBV cccDNA serves as the template for all viral transcripts, while integrated HBV can transcribe the 2.4 kb, 2.1 kb, and 0.7 kb transcripts [32,33]. In liver-humanized FRG mice infected with HBV at the plateau stage of viral titer, Peng et al. showed that over 99% of viral transcripts were derived from cccDNA. Quantitative analysis of the 3.5 kb and 2.1 kb transcripts by 5′ RNA sequencing of hepatocytes from these mice suggested that these two transcripts exhibited coordinated expression at the single-cell level [22]. Similarly, in patients with a highly active HBV infection, Gustaf et al. reported that the viral transcripts for HBsAg and HBcAg were correlated [34].

Previous studies have shown that HBV-infected hepatocytes contain only a few cccDNA molecules [13,35,36]. In a given cell containing two or more cccDNA, whether the cccDNA molecules support transcription from all promoters simultaneously or a cccDNA only supports transcription from a subset of its promoters while the generation of all transcripts needs different cccDNA is a question important for understanding the dynamics of HBV infection [36,37,38]. By using sequence alignment of viral transcripts from the cccDNA of different HBV genotypes (A/D), Peng et al. demonstrated that, in individual hepatocytes from liver-humanized FRG mice infected with both genotypes, cccDNA molecules could transcribe, from separate molecules, either all viral transcripts or transcripts primarily from the X domain without a significant expression of structural genes. Particularly in actively infected cells, different cccDNA molecules showed a highly coordinated pattern of viral transcription at the single-cell level [22]. The exact mechanism by which cccDNA achieves coordinated transcription is unclear. Kornyeyev et al. reported that the HBx protein is diffusely distributed throughout the nucleus without a discernible localization pattern, suggesting that the diffused HBx may coordinate productive transcription across different cccDNA molecules within a cell [39].

Despite the apparent coordination of cccDNA transcription in single cells, human liver samples, especially those from HBeAg-negative patients, reveal distinct or even exclusive expression patterns of HBsAg and HBV core protein in infected cells [40,41,42]. Abhishek et al. used a four-plex immunofluorescence assay to analyze biopsies from both HBeAg-positive and HBeAg-negative patients [42]. They observed diffuse staining patterns of individual HBV core-positive cells and foci of HBsAg-positive cells, with hepatocytes positive for both HBV core and HBsAg being rare. Notably, HBeAg-negative patients exhibited a predominance of HBsAg-positive/HBV core-negative cells, with HBsAg staining indicating patches of clonal expansion. This phenomenon is likely due to HBsAg originating predominantly from integrated HBV [33,42].

### 2.3. Transcription of Inactive cccDNA and Implications for Therapy

In patients with chronic HBV infection, some individuals exhibit low-level viremia or enter a state known as occult HBV infection (OBI), characterized by low levels of HBV cccDNA in hepatocytes and minimal or undetectable HBV RNA transcription [43,44]. Transcriptionally inactive cccDNA has also been identified in patients who also had HIV and had undergone long-term treatment with NUCs, as evaluated through droplet digital PCR (ddPCR) using single-cell laser capture microdissection (scLCM) [19,20,23]. Balaagopal et al. quantified cccDNA and pgRNA in approximately 2080 single hepatocytes, finding that longer NUC treatment durations often correlated with a higher proportion of hepatocytes exhibiting inactive viral transcription [20]. In line with these studies, Kim et al. found that the pgRNA transcripts became undetectable after 24 weeks of TAF therapy; however, the S and X transcripts showed no significant changes [45]. Notably, Thio et al. reported that, among persistently HBeAg-negative patients with HBV, 23% to 56% of the infected hepatocytes were viral transcription inactive, a proportion that was higher than that observed in HBeAg-positive patients with HBV. Furthermore, the percentage of viral transcription active cells significantly declined following NUC treatment [23]. However, the discontinuation of NUC treatment typically results in a viral rebound, even in patients who had been on the treatment for years and showed no detectable HBV in their blood before cessation [3,46]. The kinetics of viral rebound vary among patients, indicating different patterns of viral replication and spread within the liver.

The existence of transcriptionally inactive cccDNA has significant implications for clinical practice and drug development. Most current investigational drugs, including the most advanced candidates, result in viral relapse in the majority of patients after treatment cessation [47,48,49]. However, assessing transcriptionally inactive or non-productive, latent cccDNA and its reactivation in human patients remains challenging mainly due to the limited availability of liver biopsy samples and the shortage of technology for dissecting the latent cccDNA with minimal or no structural gene transcription. In animal studies, Allweiss et al. demonstrated that suppressed cccDNA transcription, marked by the presence of Smc5/6 in the infected cells, could be maintained by blocking viral entry [50]. Also, in liver-humanized mice, by using 5′ single-cell sequencing of HBV transcripts, Peng et al. recently showed a small proportion of hepatocytes harboring cccDNA with only low levels of HBx-related RNAs from the HBV X/enhancer I domain but no transcripts for HBV structural genes. After 4 weeks of combined treatment with a neutralizing anti-preS1 antibody and PEG-IFNα, the ratio of these non-productive hepatocytes increased from 16% to 32%, accompanied by decreased levels of viral structural genes [22].

As previously mentioned, the proportion of infected cells containing transcriptionally inactive cccDNA increases during NUC treatment. However, even under NUC treatment, viral amplification and spread are only suppressed to a low but non-negligible level [51,52]. Considering the quasispecies nature of HBV infection, cross-activation of cccDNA within individual cells may still occur, generating infectious viruses even if the original variants alone are defective. This continuous infection–reinfection cycle may be a key mechanism of persistent infection. Indeed, the cross-activation of HBV variants has been observed in cell cultures [22]. HBx gene expression from the integrated viral genome may also facilitate reactivation [32]. Given that hepatocyte renewal is highly dependent on ploidy, with diploid cells showing a more than seven-fold higher annual birth rate compared with polyploid cells [24], inactive cccDNA residing in polyploid hepatocytes may persist long term and serve as a source of reactivation. Blocking the continuous cycle of HBV infection in the liver could alter the course of chronic hepatitis B (CHB), but further clinical studies are needed to determine how best to combine various therapies for different populations of patients with HBV. Given the added divergence in HBV-infected hepatocytes, including changes in metabolism and gene expression, antiviral interventions such as entry inhibitors, which are free from concerns about membrane transportation and intracellular pharmacological differences, may be particularly suited for combination therapies aimed at shortening the duration of curative treatment.

## 3. New Research Tools for Future Studies

Despite significant progress in single-cell transcriptomic research on HBV, many questions remain unanswered. New technologies are under development but have already begun to offer fresh insights into HBV infection. Some of these include the following:

### 3.1. Spatial Transcriptomics

Hepatocytes exhibit spatial heterogeneity and zonation along the portal–central axis within the liver lobule, with both transcriptomic and proteomic zonation observed in liver cells [28,29]. Integrating spatial information to analyze the distribution and interactions between different cell types during HBV infection could lead to a better understanding of the crosstalk between HBV and host cells [53].

### 3.2. Long-Read Sequencing Technologies

HBV transcripts contain splice variants, and methods such as ddPCR or 5′ sequencing, which focus on short fragments, are limited in their ability to quantitatively assess viral transcript variants from cccDNA or integrated DNA in single cells. Long-read sequencing technologies enable a more precise analysis of viral RNA structures and complex gene regulatory networks [33].

### 3.3. Multi-Omics Integration

Integrating single-cell DNA sequencing, transcriptomics, and proteomics could enhance our understanding of the molecular mechanisms underlying HBV infection [41,42]. For example, multi-omics analysis may provide deeper insights into the nature of HBsAg-negative/HBV core-positive hepatocytes in patients [42].

### 3.4. Deep Learning

Omics and multi-omics studies generate vast amounts of data. Advanced data analysis approaches, such as deep learning, could accelerate the extraction of valuable insights from these large datasets [54,55,56], driving forward our understanding of HBV infection.
